# Content validity of a sleep numerical rating scale and a sleep diary in adults and adolescents with moderate-to-severe atopic dermatitis

**DOI:** 10.1186/s41687-020-00265-y

**Published:** 2020-11-23

**Authors:** Carla Dias-Barbosa, Rodolfo Matos, Margaret Vernon, Colleen E. Carney, Andrew Krystal, Jorge Puelles

**Affiliations:** 1 Evidera, The Ark, 201 Talgarth Road Hammersmith, London, W6 8BJ UK; 2 Evidera, 7101 Wisconsin Avenue, Suite 1400, Bethesda, MA 20814 USA; 3 Ryerson University, 350 Victoria Street, Toronto, ON M5B 2K3 Canada; 4 University of California, San Francisco, Weill Institute for Neurosciences, 401 Parnassus Avenue, San Francisco, CA 94143-0984 USA; 5 Galderma, World Trade Center, Avenue Gratta-Paille 2, 1018 Lausanne, Switzerland

**Keywords:** Atopic dermatitis, Sleep, Patient-reported outcomes, Content validity

## Abstract

**Background:**

The intense itching associated with atopic dermatitis (AD) often causes patients to experience severe sleep disturbance. Here, we describe the results of a two-phase concept elicitation and cognitive interview study to establish the content validity of a sleep disturbance numerical rating scale (SD NRS) and a Consensus Sleep Diary adapted for adults and adolescents with moderate-to-severe AD (CSD-AD©).

**Results:**

In phase I, a concept elicitation conducted in 20 adults and 10 adolescents with moderate-to-severe AD revealed that the following sleep-related issues were important and relevant: nighttime awakening (87%), trouble falling asleep (73%), feeling unrested (53%), daytime fatigue or sleepiness (53%), and feeling as if they did not get enough sleep (33%). The frequency and extent of sleep disturbance varied substantially from day to day due to varying degrees of itching and flares, medication use, and changes in the weather. All participants understood the SD NRS question, with most finding it easy or very easy to understand (100% of adults and 90% of adolescents) and most understanding the anchors as intended (95% of adults, and 100% of adolescents). Most participants (94% of adults, and 90% of adolescents) indicated that they would consider a one- or two-point change meaningful on the SD NRS. The CSD-AD© was revised based on participant feedback, and tested during phase II in a convenience sample of six adults and four adolescents from phase I. The changes made to the CSD-AD© were confirmed to be relevant and understandable. All patients were able to provide an answer to each item in the CSD-AD©, and most were able to estimate the duration of nighttime awakenings, daytime naps, and dozing.

**Conclusions:**

The study supported the content validity of the SD NRS and CSD-AD© in adults and adolescents with moderate-to-severe AD. It also emphasized the importance of using these instruments daily when assessing the benefit of a new treatment on sleep quality in this population.

**Supplementary Information:**

**Supplementary information** accompanies this paper at 10.1186/s41687-020-00265-y.

## Background

Atopic dermatitis (AD) is a common relapsing inflammatory skin condition characterized by pruritus, erythema, and lichenified skin lesions [1]. AD usually appears in childhood and, in most cases, improves with age. However, in about one in five patients, it persists into adulthood [1]. AD is thought to be caused by skin barrier dysfunctions that lead to increased immune reactions and inflammation [1, 2].

The intense itching associated with AD often causes patients to experience severe sleep disturbance, leading to daytime sleepiness and sleep-related impairment [1, 3–5]. Compared to patients with AD who do not report sleep disturbance, those who report sleep disturbance more often miss work, have doctor visits, and experience difficulties performing daily tasks [6, 7]. Sleep disturbance is reported by 33% to 87% of patients with AD [3], and as few as one in five patients report having good or very good sleep quality [5].

Although sleep disturbance is a significant problem for patients with AD, clinical trials examining AD treatments have usually focused on physician-assessed outcomes [3, 8]. Reliable tools for assessing sleep disturbance from the patient perspective are lacking [3]. Simple concepts such as itch or pain are often assessed using single-item questionnaires, whereas complex concepts such as sleep disturbance require a more detailed approach to capture the multidimensionality and daily variability of the concept. Thus, a single-item scale, such as a single NRS administered daily, is not sufficient to adequately capture the multidimensionality of the sleep disturbance concept. However, combined with a multi-item diary, it could be an appropriate approach for assessing the benefit of an intervention.

Actigraphy and polysomnography have been used to quantitatively measure objective aspects of sleep [9, 10], but they do not capture how individuals feel or function in daily life, cannot examine the effect of an intervention from the patient perspective, often do not correlate well with subjective assessments of sleep using diaries, and, in the case of actigraphy, have limited usefulness for assessing sleep onset latency and duration of awakenings [8, 11]. Several patient-reported outcomes (PROs) for assessing sleep are available. These include the Pittsburgh Sleep Quality Index for adults [3] and the PROMIS Sleep Disturbance and sleep-related impairment item banks [5]. Several other instruments are available for assessing sleep quality in children, adolescents, and adults [12, 13]. However, these instruments were not developed in consultation with AD patients, and, therefore, cannot be assumed to be fit-for-purpose or adequate for them.

According to best practices for clinical research and regulatory requirements, PROs should be consistent with the patients’ experiences and measure concepts that are clinically relevant and important to them to be considered to have content validity [14–16]. This involves first establishing the relevance of the specific concepts measured in the PRO and then testing the PRO in the intended population to ensure that the instructions are clear and the content of each question, response scale, and recall period are correctly interpreted and understood [14, 16, 17]. In the current study, qualitative data were collected to document the importance and relevance of sleep disturbance in adolescents and adults with moderate-to-severe AD and to establish the content validity of a one-item sleep disturbance numerical rating scale (SD NRS) and a version of the Consensus Sleep Diary [11] adapted to patients with AD (CSD-AD©). To aid the interpretation of the SD NRS data in clinical trials, information on what patients would consider a meaningful change was also collected.

## Methods

### Participants

Participants were identified and recruited across six clinical sites in the US (California, Florida, New York, and Texas). Participants had to be aged ≥12 years; have a clinical diagnosis of moderate-to-severe AD, as defined by an Eczema Area and Severity Index (EASI) ≥12 within 2 weeks of study enrollment [11]; and have a score ≥ 4 on the SCORing Atopic Dermatitis (SCORAD) sleep loss visual analog scale (VAS) [18] within 2 weeks of study enrollment. Participants also had to be able to read and understand English sufficiently to participate in a telephone interview and complete the assessments. Participants were excluded if they had a significant speech impairment, cognitive impairment, hearing difficulty, visual impairment, or severe psychopathology in the opinion of the site’s clinical staff. Efforts were made to recruit a diverse sample of patients that included adolescents (12–17 years), young adults (18–30 years), middle-aged adults (31–45 years), and mature adults (≥46 years).

### Phase I

Phase I consisted of a hybrid concept elicitation and cognitive debriefing interview phase to provide evidence to support the content validity of the SD NRS and CSD-AD©. Phase I included 20 adults and 10 adolescents with moderate-to-severe AD, moderate-to-severe pruritus, and sleep disturbance. Participants completed the original versions of the CSD-AD© and SD NRS and were interviewed about the instruments during one-on-one, semi-structured telephone sessions.

Evidence on content validity was gathered through concept elicitation exploring patient experiences, with the objective of determining whether the SD NRS measured a concept of relevance and importance to patients with AD. Concept elicitation was followed by cognitive debriefing to assess whether the participants fully understood the SD NRS and to determine how easily they could complete the SD NRS. Specific probes were used to discuss information not spontaneously reported by participants (e.g. frequency, severity, and duration of sleep disturbance). If a participant did not spontaneously report a concept covered by the CSD-AD, they were probed to determine its relevance. Participants’ perspectives of meaningful change thresholds for the SD NRS and “no or minimal” sleep disturbance were also elicited by asking participants what change from the current day’s score they would consider to be the smallest improvement that they would be satisfied with and what number on the scale they would consider a meaningful improvement. Participants were also asked what score on the SD NRS they would consider as indicating no or minimal sleep disturbance. The information collected was used to assess and refine the content of the CSD-AD©. Based on participant feedback from phase I, the CSD-AD© was modified and tested in phase II.

### Phase II

During phase II, a convenience sample of 10 participants from phase I completed the revised version of the CSD-AD©. These participants were interviewed about the instruments during one-on-one, semi-structured telephone sessions to examine the content validity of the CSD-AD© and ensure that any revisions were relevant, comprehensive, and understandable.

### SD NRS

The SD NRS was a single-item, self-reported 11-point scale ranging from zero to 10 for reporting the degree of sleep loss related to AD.

### CSD-AD©

The CSD-AD© was adapted from the Consensus Sleep Diary, a standardized, prospective tool for tracking nightly subjective sleep that was developed by experts and refined with patient input [11]. The Consensus Sleep Diary contains core items for assessing insomnia that are not modifiable, but it allows for a variety of optional items to be included. For the CSD-AD©, items that related to AD specifically were added, and some items were modified to be relevant for this population and to capture sleep disturbance attributed to AD symptoms. The original version of the CSD-AD© used in phase I consisted of nine items to be completed in the morning assessing concepts related to time until sleep onset, nighttime awakenings, total sleep time, and sleep quality (items 1–9), and two items to be completed in the evening assessing daytime naps and dozing (items 10a and 10b) (Table 1). For phase II, the CSD-AD© was revised based on participant feedback from phase I. The revised version of the CSD-AD© used in phase II included 11 items to be completed in the morning that assessed concepts related to disrupted nighttime sleep and sleep quality (morning items 1–11) and four items to be completed in the evening that assessed daytime naps and dozing (evening items 1–4). No further changes were made following phase II.

**Table 1:** Items Included in the Original and Revised Versions of the CSD-AD© and SD NRS

Instrument	Original Version (used in phase II)	Revised/Final Version	Change Made
**CSD-AD**©	Item 1: What time did you get into bed?	Morning item 1: What time did you get into bed?	No change
	Item 2: What time did you try to go to sleep?	Morning item 2: What time did you try to go to sleep?	No change
	Item 3: How long did it take you to fall asleep?	Morning item 3: How long did it take you to fall asleep?	No change
	Item 4a: How many times did you wake up, not counting your final awakening?	Morning item 4: How many times did you wake up due to the symptoms of atopic dermatitis (for example itching, burning), not counting the final time you woke up for the day?	Added “due to the symptoms of atopic dermatitis (for example itching, burning)” Replaced “final awakening” by “final time you woke up for the day” Examples provided in the instructions adjusted accordingly
	Item 5b: In total, how long did these itch-related awakenings last?	Morning item 5: In total, how long did the awakenings related to the symptoms of atopic dermatitis (for example itching, burning) last?	Added “awakenings related to the symptoms of atopic dermatitis (for example itching, burning)”
	Item 4b: How many times did you wake up due to itching, not counting your final awakening?	Morning item 6: How many times did you wake up, for other things (for example to drink water, to go to the bathroom), not counting the final time you woke up for the day?	Added “for other things (for example to drink water, to go to the bathroom)” Replaced “final by “final time you woke up for the day”
	Item 5a: In total, how long did these awakenings last?	Morning item 7: In total, how long did these awakenings related to other things (for example to drink water, to go to the bathroom) last?	Added “for other things (for example to drink water, to go to the bathroom)”
	Item 6: What time was your final awakening?	Morning item 8: What time did you wake up for the day?	Replaced “final awakening” by “time did you wake up for the day”
	(No item)	New morning item 9: What time did you get out of bed for the day?	Insertion of item 7 from the Consensus Sleep Diary [11] to be able to derive sleep metrics
	Item 7: How would you rate the quality of your sleep?	Morning item 10: How would you rate the quality of your sleep?	No change
	Item 8: How rested or refreshed did you feel when you woke up for the day?	Morning item 11: How rested or refreshed did you feel when you woke up for the day?	No change
	Item 9: Did you take any sleep medication last night?	(Deleted)	Removed because sleep medications are usually not allowed in clinical trials of atopic dermatitis
	Item 10a: How many times did you nap or doze? Item 10b: In total, how long did you nap or doze?	Evening item 1: How many times did you nap?	No change; broken down into separate questions
		Evening item 2: In total, how long did you nap?	
		Evening item 3: How many times did you doze off?	
		Evening item 4: In total, how long did you doze off?	
**SD NRS**	In instructions, atopic dermatitis abbreviated “AD”	In instructions, “atopic dermatitis” spelled out	Changed from “AD” to “atopic dermatitis”
	Anchor for the maximum score (10) defined as “I cannot sleep at all”	Anchor for the maximum score (10) defined as “I did not sleep at all”	Changed from “cannot” to “did not”

*Abbreviations*: *CSD-AD©* Consensus Sleep Diary, atopic dermatitis version, *SD NRS* sleep disturbance numerical rating scale

### Clinician-reported outcomes

The EASI is a clinician-reported scale designed to measure the severity and extent of AD [19]. The EASI is a composite score based on the total area affected and intensity of redness, thickness, scratching, and latensification on the head/neck, trunk, upper limbs, and lower limbs. The score ranges from 0 to 72, with a higher score indicating more severe AD.

SCORAD is a hybrid clinician- and self-reported tool that includes the extent, intensity, and symptoms of AD [18]. The SCORAD total score ranges from 0 to 103, with a higher score indicating more severe AD. Included in SCORAD are VASs in which sleeplessness and pruritus are scored from 0 for “none” to 10 for “worst possible.”

### Interviews

The interviews followed the methodology of the US Food and Drug Administration and ISPOR for developing PRO instruments [15, 16, 20]. All interviews were conducted in US English by trained qualitative researchers. Clinical site investigators were responsible for ensuring that all participants fully understood the nature and purpose of the interview. Potential participants received a consent form describing the details of the study, which they reviewed with the site’s clinical staff, and were given the opportunity to ask questions about the study.

After providing informed consent, each participant was sent an interview pack that contained the CSD-AD© and SD NRS in a sealed envelope. Participants were asked not to open the envelopes until the time of the interview session. They were asked to return the completed questionnaires after completion of the interview. Demographic and clinical data, EASI scores, and the SCORAD were collected from eligible, consenting participants by the site’s clinical staff.

Experienced and trained staff conducted interviews by telephone using semi-structured interview guides. Interviewers were trained in study-specific objectives and the sponsor’s adverse event reporting requirements.

The discussion guides began with open-ended questions that were followed by semi-structured interviews. The discussion guides included probes for interviewers to obtain detailed information on specific issues not spontaneously reported by the participants. For the concept elicitation portion, participants were asked open-ended questions about their experiences with AD and sleep disturbance. The patients’ perspectives on meaningful change in the SD NRS were probed, including the smallest improvement with which the participants would be satisfied or content, the level of improvement that they would consider meaningful, and what they would consider a “no or minimal” sleep disturbance. For the cognitive debriefing portion, participants were asked questions about the comprehension, relevance, and acceptability of the SD NRS and CSD-AD©.

All identifying information was removed from the transcripts to maintain the confidentiality of all protected health information. Interviews lasted approximately 90 min and were audio recorded.

### Data management and quality control

Audio-recorded data collected during the interviews were transcribed by third-party professional transcription services. Audio files from the interviews were reviewed by the scientific staff for quality assurance purposes to remove public health information and correct obvious transcription errors. Quantitative sociodemographic and clinical data were transmitted using a secure file transfer portal directly into the electronic system database. An electronic image of the case report form was then entered into the database and reviewed by project scientific staff. Data discrepancies were identified and resolved.

The scientific staff was responsible for the overall direction and supervision of the data collection, as well as for monitoring the study progress and quality control of data. All work was subject to quality control, as well as documentation procedures to ensure that the data were accurate, and the analyses could be reproduced.

### Data analysis

Sociodemographic and clinical characteristics collected in phase I were characterized using descriptive statistics for the entire participant sample. Quantitative analyses were performed using SAS version 9.4 (SAS Institute, Cary, NC, USA) [21].

Qualitative data collected in transcripts were analyzed using a content analysis approach. The coding process was driven by the objectives of the study and consisted of tagging codes to segments of textual data to facilitate the comprehension of a large amount of data. Concept codes were used to capture the participants’ descriptions of their experiences with sleep problems, and the impact on their everyday life. Specific codes related to comprehension, relevance, and acceptability of the items, instructions, response options, recall periods, and ease of use/completion of the measures were also used. All qualitative analyses were performed using ATLAS.ti, version 7.0 or higher (Scientific Software Development GmbH, Berlin, Germany) [22]. Coding dictionaries were developed to aid with the coding. Relevant concepts mentioned in the interviews were tracked to monitor the saturation of concepts. Saturation was defined as the point at which no substantially new information or concepts continued to emerge beyond what had been mentioned in previous interviews [23, 24]. Saturation was documented with the help of saturation grids, where concepts (spontaneously reported or probed) endorsed by participants were listed vertically, and study participant identification numbers representing each individual interview were listed horizontally [14]. The qualitative interviews in phase I were also used to identify thresholds for a meaningful change, and the thresholds for “no or minimal” sleep disturbance in the SD NRS.

### Ethics

Prior to participant recruitment, institutional review board approval (Advarra, Columbia, MD) of the study protocol was obtained. All recruitment procedures complied with current Health Insurance Portability and Accountability Act regulations in the US. Adult participants had to provide written informed consent prior to study procedures. Adolescent participants had to provide informed assent, and their parent or legal guardian had to provide written permission for their child to participate beforehand. All participants also had to consent to being audio recorded during the discussions.

## Results

### Phase I

#### Participants

In phase I, 20 adult and 10 adolescent participants were enrolled between December 2018 and April 2019 across six clinical sites in the US. The mean age of the adult participants was 33.5 years, and the majority were female (*n* = 12). The mean age of the adolescent participants was 14.1 years, and equal numbers were male and female (*n* = 5 each) (Table 2). Most participants were White or Asian, and most identified themselves as not Hispanic. According to the site’s clinical staff, pruritus was severe for most participants (*n* = 13 adults, *n* = 7 adolescents); all other patients were described as having moderate pruritus. The mean ± standard deviation (SD) Eczema Area and Severity Index (EASI) score was 25.7 ± 11.5 (median [range] = 22.5 [12.0–50.9]) for adults and 26.3 ± 8.9 (median [range] = 25.0 [12.2–38.5) for adolescents. The mean ± SD SCORing Atopic Dermatitis (SCORAD) score was 72.5 ± 11.0 (median [range] = 70.8 [57.0–97.4]) for adults and 78.6 ± 15.6 (median [range] = 75.0 [52.0–95.8]) for adolescents. The EASI and SCORAD scores indicate that all patients had moderate to severe AD [25].

**Table 2:** Participant Demographics and Clinical Characteristics

Characteristic	Phase I		Phase II	
	Adults (***N*** = 20)	Adolescents (***N*** = 10)	Adults (***N*** = 6)	Adolescents (***N*** = 4)
**Age (Years), Mean ± Standard Deviation**	33.5 ± 12.8	14.1 ± 1.9	30.3 ± 8.7	14.0 ± 2.2
**Sex, n (%)**				
Male	8 (40)	5 (50)	5 (83)	2 (50)
Female	12 (60)	5 (50)	1 (17)	2 (50)
**Ethnicity, n (%)**				
Hispanic or Latino	5 (25)	2 (20)	1 (17)	2 (50)
Not Hispanic or Latino	15 (75)	8 (80)	5 (83)	2 (50)
**Racial Background, n (%)**				
White	8 (40)	4 (40)	2 (33)	1 (25)
Black or African American	4 (20)	0 (0)	2 (33)	2 (50)
Asian	7 (35)	5 (50)	2 (33)	0 (0)
American Indian or Alaska Native	1 (5)	0 (0)	0 (0)	0 (0)
Other	0 (0)	1 (10)	0 (0)	1 (50)
**Pruritus**^**a**^**, n (%)**				
Mild	0 (0)	0 (0)	0 (0)	0 (0)
Moderate	7 (35)	3 (30)	2 (33)	1 (25)
Severe	13 (65)	7 (70)	4 (67)	4 (75)
**EASI**				
Mean ± Standard Deviation	25.7 ± 11.5	26.3 ± 8.9	33.6 (8.4)	31.1 (8.0)
Median [Range]	22.5 [12.0–50.9]	25.0 [12.2–38.5]	32.8 [23.1–48.1]	32.0 [21.8–38.5]
**SCORAD**				
Mean ± Standard Deviation	72.5 ± 11.0	78.6 ± 15.6	71.7 (6.7)	82.6 (12.3)
Median [Range]	70.8 [57.0–97.4]	75.0 [52.0–95.8]	70.8 [63.9–80.4]	82.6 [73.9–91.3]
**SCORAD Pruritus VAS**				
Mean ± Standard Deviation	7.7 ± 1.6	7.6 ± 1.7	5.9 (1.3)	7.9 (1.7)
Median [Range]	8.1 [4.0–9.8]	8.0 [5.4–10.0]	6.2 [4.0–7.1]	7.9 [6.7–9.1]
**SCORAD Sleep Loss VAS**				
Mean ± Standard Deviation	7.2 ± 1.6	6.7 ± 1.9	5.5 (1.6)	6.0 (0.8)
Median [Range]	7.5 [4.0–9.7]	6.5 [4.3–10.0]	5.2 [4.0–8.1]	6.0 [5.4–6.5]

*Abbreviations*: *EASI* Eczema Area and Severity Index, *SCORAD* Scoring Atopic Dermatitis, *VAS* visual analog scale

^a^According to the site’s clinical staff

The mean ± SD SCORAD VAS for pruritus was 7.7 ± 1.6 (median [range] = 8.1 [4.0–9.8]) for adults and 7.6 ± 1.7 (median [range]: 8.0 [5.4–10.0]) for adolescents. The mean ± SD SCORAD VAS for sleep loss was 7.2 ± 1.6 (median [range] = 7.5 [4.0–9.7]) for adults and 6.7 ± 1.9 (median [range] = 6.5 [4.3–10.0]) for adolescents. Thus, all patients had moderate to very severe pruritus and sleep loss.

#### Concept elicitation

**Fig. 1:**
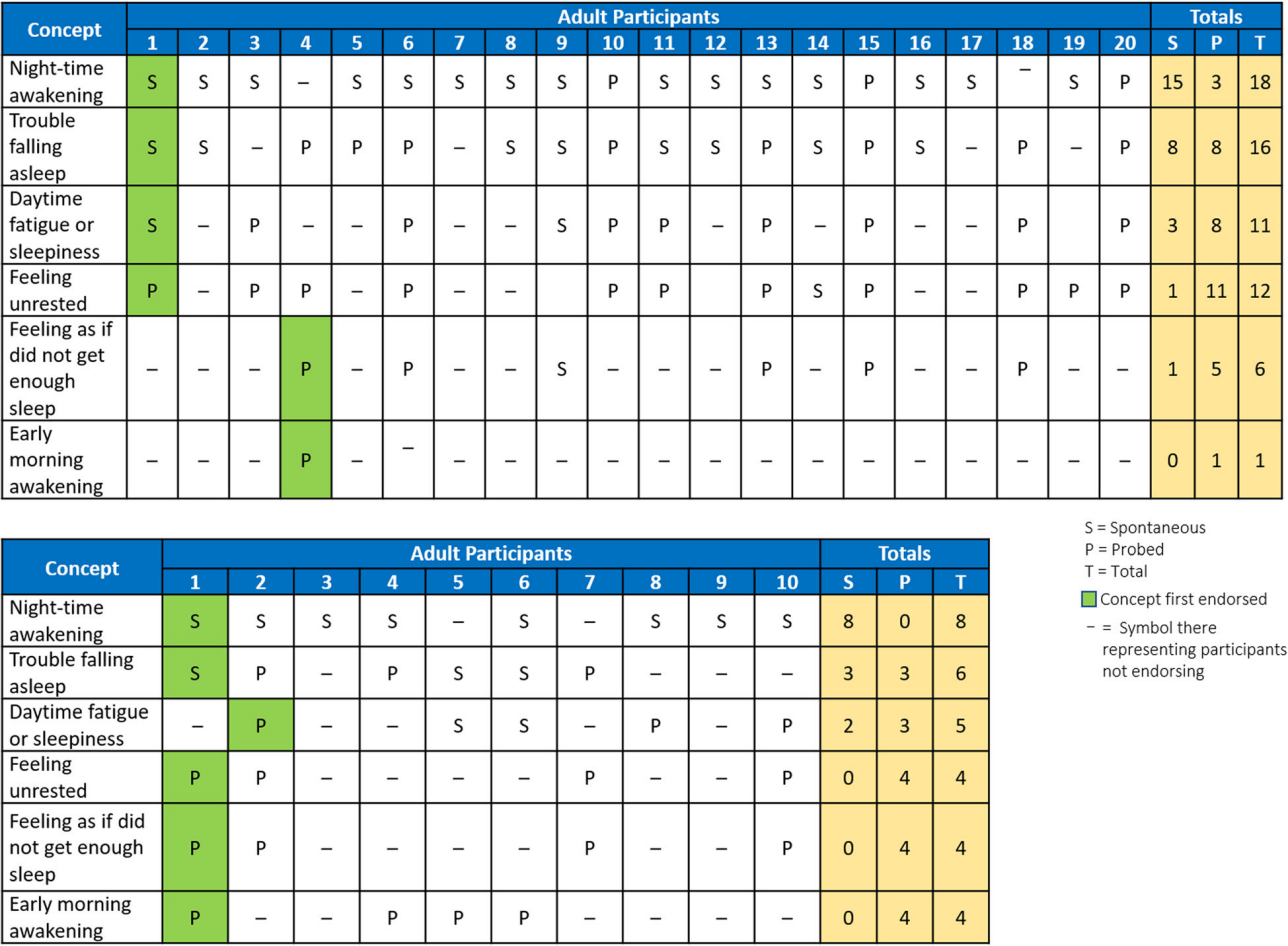
Saturation Concepts by Individual Adult and Adolescent Participants

Data saturation was assessed following an inductive thematic approach after all interviews had been completed [23, 24]. Figure 1 shows the saturation grids that summarize the concepts that emerged during the interviews with adults and adolescents, as well as the number of participants who mentioned sleep disturbance concepts spontaneously versus when probed. Saturation was reached after four adult participant interviews and two adolescent participant interviews.

#### Description of sleep disturbance

Overall, the most frequent sleep-related issue reported was nighttime awakening (87% overall: 80% of adults and 60% of adolescents), followed by trouble falling asleep (73% overall: 90% of adults and 80% of adolescents), feeling unrested (53% overall: 60% of adults and 40% of adolescents), daytime fatigue or sleepiness (53% overall: 55% of adults and 50% of adolescents), and feeling as though they had not gotten enough sleep (33% overall: 30% of adults and 50% of adolescents) (Fig. 1 and Table 3). Early morning awakening was mentioned by 40% of adolescents, but reported by only 5% of adults.

**Table 3:** Phase I Concept Elicitation Results

	Frequency		Illustrative Quotes	
Concept	Adults (***N*** = 20)	Adolescents (***N*** = 10)	Adult participants	Adolescent participants
**Description of Sleep**			*“I have problems falling asleep, I wake up several times during the night mainly due to itching …*”	*“Sometimes I wake up in the night, but once I get to bed I kind of sit there because my body gets itchy and I have to scratch it a bit before I sleep and then sometimes, I wake up”*
Nighttime awakenings	90%	80%		
Trouble falling asleep	80%	60%		
Feeling unrested	60%	40%		
Daytime fatigue or sleepiness	55%	50%		
Did not get enough sleep	30%	40%		
Early-morning awakening	5%	40%		
**Frequency of Sleep Disturbance**			*“… when it’s a bad flare … it’s pretty much nightly … when I’m relatively healthier and my skin condition is kind of being managed better, then I really don’t wake up as much”*	“… *it’s definitely in the summer and winter because of the dramatic weather changes”*
Daily	70%	30%		
Less than daily	20%	70%		
Did not specify	10%	–		
**How Sleep Disturbance Varied** ^**a**^			*“… it depends on how bad and how broad it covers. So, if it’s just in one section, it’s not as bad as sometimes I have it everywhere and it’s really hard for me to fall back asleep because there’s no comfortable position, it’s itching everywhere”*	*“… one night I might sleep fine, wake up and like itch my knee or something and fall right back to sleep. Then some nights I might wake up three times and be itching a lot and not really sleep that much”*
Depended on flare ups	32%	20%		
Depended on medication use	16%	20%		
Depended on weather	5%	–		
Varied from day to day but did not provide reason	26%	60%		
Depended on severity or extent of itching	5%	–		
Sleep did not vary	16%	–		
**Duration of Sleep per Day**			*“I probably only get like maybe 4 h of sleep at night, if that. And then with the naps, it’s probably I’ll only sleep 6 h a day, seven maybe. But on the weekends, I sleep more obviously because I can take a nap whenever I want.”*	“*… around 6–8 h”*
3 to 4 h	5%	10%		
4 to 8 h	90%	80%		
> 8 h	5%	10%		
**Awakenings per Night**			*“… sometimes I’ll sleep the whole night through. Then sometimes I’ll wake up as many as like four time.”*	“*Usually, I usually wake at least two times a day, so it’s kind of easy to me now to remember how many times I wake up”*
None	5%			
1 to 2	10%	40%		
2 to < 10	75%	60%		
Up to 10	5%			
Unknown	5%			
**Ease of Falling Back Asleep after Nighttime Awakenings** ^**b**^			*“Depending on how bad I scratch or how itchy it is.”*	*“Sometimes … I just get up and I regather myself and I fall back to sleep. But sometimes it’s hours before I can actually finally go back to sleep”*
Easily	44%	67%		
Sometimes easily	31%	17%		
Difficulty	25%	17%		
**Reasons for Sleep Disturbance**			*“… the main causes would be eczema and just how bad the flare up is … when my skin is really bad, I get really anxious that I’m going to be scratching all night …”*	*“[Main cause of sleep disturbance is panic attacks] correlated to when I’m itchy.”*
Itching from AD	35%	30%		
Itching from AD + other causes	65%	70%		
**Impact on Daily Life**			*“… I feel always drowsy in the morning …*. *after 2:00 p.m., yeah, I feel really tiring, so I have to take a nap for 30 min at least …”*	*“I just feel like I’m not performing to my best because I’m drowsy or not paying full attention because I’m tired or I’m thinking about something else just because my head is not fully there.”*
Feeling tired, fatigued, or drowsy during the day	95%	50%		
Impact on work or school	30%	60%		
Affects mood, increased irritability	20%			
No effect	15%	10%		

*Abbreviation*: *AD* atopic dermatitis

^a^ *N* = 19 adults, *N* = 10 adolescents

^b^ *N* = 16 adults, *N* = 6 adolescents

#### Frequency of sleep disturbance

Sleep disturbance was daily for 70% of adults and 30% of adolescents, less than daily for 20% of adults and 70% of adolescents, and not specified for 10% of adults. How sleep disturbance varied was obtained from 19 adults and 10 adolescents. For several patients (32% of adults and 20% of adolescents), variation in sleep disturbance was related to whether they were experiencing a flare up. Several others (16% of adults and 20% of adolescents) indicated the sleep disturbance depended upon whether or not they had used their medication for AD that day, and one adult participant (5%) stated that their sleep disturbance depended on the weather. Other participants (26% of adults and 60% of adolescents) described the extent of variation in sleep disturbance but did not provide reasons. A few adults (16%) indicated that the severity of their sleep disturbance did not vary from day to day.

#### Duration of sleep disturbance

The duration of total daily sleep varied between participants. One adult (5%) reported sleeping as little as three to 4 h per day, while another (5%) reported sleeping eight to 9 h per day. One adolescent (10%) described sleeping as little as 4 h per day, while another (10%) slept eight to 10 h. All other participants reported durations of total daily sleep between these extremes.

#### Description of nighttime awakenings

The number of awakenings each night varied greatly, although most adult and all adolescent participants reported 1–10 awakenings per night. Two adult participants (10%) reported waking up 1–2 times per night, and one (5%) reported that they did not usually wake up during the night because of being too tired. On the extreme end, another adult (5%) estimated waking up as many as 10 times per night. A few adolescents (30%) reported waking up 2–3 times per night; others reported waking up 1–2 times per night (20%), two times per night (20%), or five times per night (20%).

The duration of awakenings also varied. One adult (5%) reported being awake for as little as a few seconds, while another (5%) would be awake for an hour. Some adults (15%) noted that the amount of time they were awake would vary from a couple minutes to an hour. One adolescent participant (10%) reported being awake for 10–15 min, while another (10%) would be awake for an hour. Another adolescent (10%) noted that the amount of time awake varied, as it would sometimes be for 15 min and other times for “hours.”

Of 16 adults asked about the ease of falling back asleep after a nighttime awakening, 44% noted that they could fall asleep easily or quickly and 31% reported difficulty falling back asleep. Some (31%) explained that it varied depending on how tired they were, how bad the itch was, or whether they got out of bed to use medication, use lotion, or to shower. Of six adolescents asked this question, most (67%) reported that they could fall back to sleep easily or quickly.

#### Causes of sleep disturbance

Several adults (37%) and adolescents (43%) attributed their sleep disturbance solely to itching from AD. The remaining participants attributed the sleep disturbance to a combination of AD-related symptoms (e.g., itching, burning sensations, inflammation, or pain) and other non-AD causes (e.g., consuming caffeinated beverages, asthma, stress, anxiety, or panic attacks).

#### Impact of sleep disturbance on quality of life

Several adults (63%) and adolescents (56%) mentioned feeling tired, fatigued, or drowsy during the day. Some adult participants (32%) discussed an impact on work, and most adolescents (67%) reported an impact on school. Some adults (11%) reported that sleep disturbance affected their mood, reporting feeling “impatient,” “snappy,” “irritable,” or not “cheery;” other effects included increased stress levels or being lazier during the day (5% each).

#### Cognitive interview results: CSD-AD©

All adolescents and 90% of adults had a favorable opinion of the CSD-AD© (Table 4). Typical comments included, “It’s actually really easy to fill out and follow,” “Simple and easy to understand,” “Overall, it’s pretty basic, easy for me to answer,” and “I think it really helped me see where I was and how I was doing.” One adult participant (5%) commented that the instrument was wordy, though straightforward; another adult (5%) reported that there were too many questions in the instrument. One more adult (5%) commented that they had a difficult time accurately quantifying his responses for several items.

**Table 4:** Phase I Cognitive Debriefing Results for the SD NRS and CSD-AD©

Measures	Assessments	Adults (***N*** = 20)	Adolescents (***N*** = 10)
**CSD-AD**©	Overall favorable opinion	90%	100%
	Recommended additional items	35%	10%
	Recommended changes to instructions	5%	0%
	Could differentiate between questions to be completed in the morning vs. evening	80%	70%
	Found examples were helpful	75%	100%
	Recommended rewording	20%	0%
**SD NRS**	Able to describe the question properly	95%	100%
	Level of difficulty answering the question		
	Easy or very easy	85%	100%
	Difficult	10%	
	Not asked	5%	
	Understood scale and anchors	90%	100%
	Comprehension of 24-h recall period	100%	100%
	Ability to describe recall period^a^		
	Could describe the 24-h period	72%	100%
	Did not describe but was ok	17%	
	Too short	6%	
	No response	6%	
	Able to recall itch in the past 24 h or recall period was appropriate		
	Yes	45%	80%
	Provided feedback about recall period	25%	
	No issue with recall period or did not provide feedback	30%	20%

*Abbreviations*: *CSD-AD©* Consensus Sleep Diary, atopic dermatitis version, *SD NRS* sleep disturbance numerical rating scale

^a^ *N* = 18 adults, *N* = 10 adolescents

Most participants (80% of adults and 70% of adolescents) reported that they could differentiate between the questions in the CSD-AD© that had to be completed in the morning and in the evening. Most adults (75%) and all adolescents stated that the examples in the CSD-AD© were helpful. A few adults (20%) commented that one or more instructions for the CSD-AD© needed improvement, and one adult (5%) expressed that the instructions needed additional clarifications. Suggestions for improving the instructions included: specifying the differences between naps and dozing, including adding “about your sleep last night” to the top of the questionnaire, and having examples explained.

#### Cognitive interview results: SD NRS

All participants understood the SD NRS question; most found it easy or very easy to understand (100% of adults and 90% of adolescents), and most understood the anchors as intended (95% of adults and 100% of adolescents) (Table 4). A few adults (25%) and adolescents (10%) provided suggestions for improving the item. Suggestions included replacing the abbreviation “AD” with “atopic dermatitis” (15% of adults and 10% of adolescents), adding “eczema” in parenthesis next to “atopic dermatitis” (10% of adults), reducing the scale from 1 to 10 to 1–5 (5% of adults), adding “getting better” next to the anchor description of “no sleep loss” (5% of adults), and adding a description under the response option for five on the scale (10% of adolescents).

#### Meaningful change in SD NRS

If sufficient time was available during the interviews, participants were queried about what they considered a meaningful change. Of participants (*n* = 19 adults and *n* = 9 adolescents) asked about the smallest improvement they would be satisfied or content with on the SD NRS, most (69% of adults and 88% of adolescents) indicated a change of one to three points, with a two-point change being the most frequent response (32% of adults and 44% of adolescents) (Table 5). Of participants (*n* = 17 adults and *n* = 10 adolescents) probed about the levels of improvement that would be meaningful, most (94% of adults and 90% of adolescents) indicated that they would consider a change of one or two points in the SD NRS a meaningful change. Of participants (*n* = 18 adults and n = 10 adolescents) asked about what they considered no or minimal sleep disturbance, the most frequent response was score of two (41% of adults and 40% of adolescents), followed by one (29% of adults and 30% of adolescents) and three (24% of adults and 10% of adolescents).

**Table 5:** Meaningful Change in SD NRS

Illustrative Quotes				
Assessment	Adults	Adolescents	Adults	Adolescents
**Smallest Improvement with which Participants Would Be Satisfied or Content**	***N*** **= 19**	***N*** **= 9**	*… any improvement is good …*	*I feel like if I was a 5, if I went down that would be cool, but getting to a 5 would be—I mean it’s like any improvement would be meaningful.*
1 point	11%	22%		
2 points	32%	44%		
3 points	26%	22%		
4 points	16%	–		
5 points	11%	–		
6 points	–	11%		
8 points	5%	–		
**Level of Improvement that Would Be Meaningful**	***N*** **= 17**	***N*** **= 10**	*[An improvement of one point]: Maybe I might sleep a little bit better or maybe I have sleep for more hours without dealing with a medication.* *[An improvement of two points] means I could go to sleep better and that means I don’t have to wake up in the middle of the night knowing that I’m scratching myself or that means I can put makeup on, and it will cover my face.*	*I just feel like the one-point difference is kind of understandable for just a weekly basis.* *[An improvement of three points] I think I would definitely recognize that, because, I mean, like it’s just a big change from waking up a couple times to being awake for the duration of the night.*
1 point	76%	80%		
2 points	18%	10%		
3 points	6%	10%		
**Participants’ ratings for “No or Minimal sleep disturbance**	***N*** **= 18**	***N*** **= 10**	*Well, if my sleep was like a one, I would say that’s still pretty good, that the itch is not there and if I wake up it’s not going to keep me up, it won’t keep me up*.	*I’m sleeping at a one or a two, and just being to sleep well because that’s like a basic thing that everybody else can do. It’s just something that I wish I had the privilege to be able to just sleep through the night without waking up from my neck hurts or thinking about how my skin is bothering. I kind of just wish I could sleep like a normal person.*
0	11%	10%		
1	29%	30%		
2	41%	40%		
3	24%	10%		
4	–	10%		

*Abbreviation*: *SD NRS* sleep disturbance numerical rating scale

#### Changes made to the CSD-AD© following phase I interviews

Changes made to the CSD-AD© following phase I interviews are summarized in Table 1. Items 4a–b and 5a–b in the original sleep diary were retained in the revised version (as morning items 4 and 7) to estimate the number and duration of awakenings due to AD symptoms and other reasons. The term “final awakening” in these items was replaced by “final time you woke up for the day” because the wording was confusing for some participants. The concept of “itch-related awakenings” in the items 4b–5b was replaced by “awakenings related to AD symptoms” (e.g., itching and burning) to cover all AD symptoms that may cause sleep disturbance. Items 10a–b in the original CSD-AD© were separated into four items in the revised CSD-AD© because they measured two different concepts within the same item (number and duration of naps or dozing). Although participants understood the concepts as intended (i.e., that dozing lasted seconds or a few minutes, whereas naps lasted many minutes to over an hour), a few (*n* = 3) had difficulty in providing a combined answer, and one participant suggested separating the two concepts. In the revised CSD-AD©, these items were separated into four different items, including different items for dozing and naps, but the wording remained unchanged. An item asking participants what time they get out of bed for the day that is included in the CSD [11] but not in the original version of the CSD-AD© was added to allow key sleep metrics to be derived, such as total time spent in bed and sleep efficiency.

#### Changes made to the SD NRS following phase I interviews

Based on the recommendations made during the cognitive interviews during phase I, the abbreviation “AD” in the SD NRS was replaced with “atopic dermatitis” or “your skin disease,” and the anchor “I cannot sleep at all” was changed to “I did not sleep at all” (Table 1).

### Phase II

#### Participants

A convenience sample of six adult and four adolescent participants from phase I were invited to participate in phase II, which examined the content validity of the revised CSD-AD©. The mean age was 30.3 ± 8.7 years for the adult participants and 14.0 ± 2.2 years for the adolescent participants (Table 2). Participants were mostly White (*n* = 2 adults and *n* = 1 adolescent), Asian (*n* = 2 adults and *n* = 2 adolescents), or Black or African American (*n* = 2 adults). Most adult participants were not Hispanic or Latino (*n* = 5), but half of adolescent participants (*n* = 2) reported being Hispanic or Latino. According to the site’s clinical staff, most participants had severe pruritus (*n* = 4 adults and *n* = 3 adolescents). The mean EASI was 33.6 ± 8.4 for adults and 31.1 ± 8.0 for adolescents, and the mean SCORAD was 7.0 ± 6.7 for adults and 82.6 ± 12.3 for adolescents. The mean SCORAD VAS for pruritus was 5.9 ± 1.3 for adults and 7.9 ± 1.7 for adolescents, and the mean SCORAD VAS for sleep loss was 5.5 ± 1.6 for adults and 6.0 ± 0.8 for adolescents.

#### Cognitive interview results

Overall, the majority of participants understood the items of the revised CSD-AD© as intended (Table 6). Participants also indicated being able to accurately recall and select an answer for each item. Only two items (morning items 5 and 7) appeared difficult to understand and recall accurately. Morning item 5, which asked how long awakenings due to AD lasted, was found by one participant to be “a little bit confusing” because of not clearly understanding that the total number of minutes was to include time awake not only directly due to itching but also as an indirect consequence of itching. In addition, one participant did not clearly understand the recall period of morning item 7, which asked how long awakenings due to other reasons lasted, asking, “Are you talking about last night specifically or sometime during the month or?” Morning items 5 and 7 were also found to be difficult to recall accurately by one participant each. In the case of morning item 5, the participant stated that the total time “… might not be too accurate because I didn’t check my phone those times.” For morning item 7, the participant found the instructions clear, but stated, “I myself can’t really be accurate toward it.”

**Table 6:** Phase II Assessment of the Revised CSD-AD©

Items	Item was Clear and Easy to Understand	Ease of Recalling the Experience			Instructions were Clear and Easy to Understand
		Easy	Somewhat Easy	Difficult	
**CSD-AD© Morning Items**					
Item 1. What time did you get into bed?	8/8 (100)	9/9 (100)	0/9 (0)	0/9 (0)	5/5 (100)
Item 2. What time did you try to go to sleep?	10/10 (100)	8/9 (89)	1/9 (11)	0/9 (0)	7/7 (100)
Item 3. How long did it take you to fall asleep?	9/9 (100)	6/10 (60)	4/10 (40)	0/8 (0)	7/7 (100)
Item 4. How many times did you wake up due to the symptoms of atopic dermatitis (for example itching, burning), not counting the final time you woke up for the day?	8/8 (100)	7/10 (70)	3/10 (30)	0/10 (0)	7/7 (100)
Item 5. In total, how long did the awakenings related to the symptoms of atopic dermatitis (for example itching, burning) last?	8/8 (100)	5/9 (56)	3/9 (33)	1/9 (11)	6/7 (86)
Item 6. How many times did you wake up, for other things (for example to drink water, to go to the bathroom), not counting the final time you woke up for the day?	8/8 (100)	6/7 (86)	1/7 (14)	0/10 (0)	7/7 (100)
Item 7. In total, how long did these awakenings related to other things (for example to drink water, to go to the bathroom) last?	7/8 (88)	6/8 (75)	1/8 (13)	1/8 (13)	6/6 (100)
Item 8. What time did you wake up for the day?	8/8 (100)	9/9 (100)	0/9 (0)	0/9 (0)	7/7 (100)
Item 9. What time did you get out of bed for the day?	8/8 (100)	10/10 (100)	0/10 (0)	0/10 (0)	7/7 (100)
Item 10. How would you rate the quality of your sleep?	8/8 (100)	9/9 (100)	0/9 (0)	0/9 (0)	7/7 (100)
Item 11. How rested or refreshed did you feel when you woke up for the day?	8/8 (100)	8/8 (100)	0/8 (0)	0/8 (0)	7/7 (100)
**CSD-AD© Evening Items**					
Item 1. How many times did you nap?	8/8 (100)	8/8 (100)	0/8 (0)	0/6 (0)	7/7 (100)
Item 2. In total, how long did you nap?	8/8 (100)	6/6 (100)	0/6 (0)	0/6 (0)	6/6 (100)
Item 3. How many times did you doze off?	7/7 (100)	7/8 (86)	1/8 (13)	0/8 (0)	7/7 (100)
Item 4. In total, how long did you doze off?	7/7 (100)	4/5 (80)	1/5 (20)	0/5 (0)	7/7 (100)

*Abbreviation*: *CSD-AD©* Consensus Sleep Diary-atopic dermatitis version

## Discussion

This qualitative study supports the importance and relevance of sleep disturbance in adolescents and adults with moderate-to-severe AD and pruritis. We found that AD-associated sleep disturbance is a multidimensional concept, and that nighttime awakenings, trouble falling asleep, and feeling unrested are experienced by most participants. Other common issues included daytime fatigue or sleepiness, a feeling of not having had enough sleep, and an early-morning awakening. The current study also revealed that the degree of sleep disturbance varies substantially from day to day in patients with AD due to varying extents of itching and flares, medication use, and changes in the weather.

Our findings agree with a recent systematic literature review, which reported that adults with AD frequently experience sleep disturbance (prevalence of 33% to 87.1%), difficulty falling asleep, frequent/extended awakenings, and shorter sleep durations, which result in daytime sleepiness, fatigue, and reduced functioning [3]. The current results also agree with studies showing that a sleep disturbance is one of the most important components of the burden of chronic itch [26], that pruritus and scratching are a principal cause of sleep disturbance in patients with AD [27], and that the degree of sleep disturbance and sleep loss corresponds with the severity of AD [4, 5].

To measure daily sleep disturbance in patients with AD, we adapted the Consensus Sleep Diary, a standardized, prospective tool for tracking nightly subjective sleep [11]. The resulting CSD-AD© effectively captured the different dimensions of the sleep disturbance concept, and the items included in the revised version were understood as intended. Participants were able to accurately recall and select an answer to each question, and they indicated that the concepts included in the CSD-AD© were important and relevant. Using the CSD-AD©, we found that one-third of participants attributed the sleep disturbance solely to their itching, while the remaining participants attributed the sleep disturbance to both AD-related symptoms (e.g. itching, burning sensations, inflammation, or pain) and other non-AD causes (e.g. consuming caffeinated beverages, asthma, stress, anxiety, or panic attacks). A critical finding was that most participants were able to distinguish between night-time awakenings due to AD-symptoms and night-time awakening due to other causes. They also indicated that they could recall the number and duration of their awakenings.

The CSD-AD© was revised based on the results of the interviews during the first phase of the study. Changes to the CSD-AD© included adding one item, separating some items into multiple, changing the item wording, changing the order of items, and modifying the instructions. One important issue was that some items measured two distinct concepts within the same item (e.g., number and duration of naps or dozing), and used a different timeframe, (daytime) requiring an additional administration of the questionnaire in the evening. Based on participant feedback, different items were created for dozing and naps. To ensure that the changes made to the CSD-AD© were endorsed and understood by the target population, during phase II, additional interviews were conducted with a convenience sample of six adult and four adolescent participants from phase I. The revised CSD-AD© was endorsed and understood by the participants, and no further changes were needed. Only two items appeared difficult to understand and recall accurately. This is unlikely to be problematic in the context of a clinical trial, where the participants will be trained on the use of the diary and where they will be instructed to report their nighttime awakenings within an hour of getting out of bed. Because the Consensus Sleep Diary was originally developed for insomnia, the scoring and psychometric properties of the CSD-AD and the additional sleep parameters derived from the new items remain to be assessed in AD.

To support the potential use of the SD NRS as an endpoint in AD clinical trials for evaluating the meaningful treatment effects on this population, we also investigated thresholds for meaningful change. Although data on meaningful change are often collected using quantitative approaches (especially anchor- and distribution-based methods) [28], we evaluated thresholds using a qualitative approach to reflect the direct patient perspective, which is increasingly valued by regulators [29]. Most participants considered a one-point improvement to be a meaningful change, although they most frequently reported that they would be satisfied or content with a two-point change. This information will be used to inform the responder definition threshold estimates assessed quantitatively using anchor- and distribution-based methods.

Additional research is in progress to generate quantitative evidence on the psychometric measurement properties of the CSD-AD© and SD NRS, as well as to derive responder definition threshold estimates. In addition, following International Society for Pharmacoeconomics and Outcomes Research principles [30, 31], we are conducting linguistic validations of the translations, migrating the questionnaires to electronic devices, and conducting usability testing of the selected devices and cognitive debriefings to assess device functionality, instrument comprehension, and ease of use in the intended population.

This study benefitted from the inclusion of a relatively large group of patients with AD who were recruited from both sexes, geographically diverse regions of the US, and different ethnic and racial backgrounds. A potential limitation of this study could be that although the CSD is intended for insomnia, the CSD-AD© was revised based on interviews of patients that were not specifically diagnosed with insomnia. Thus, the findings from the CSD-AD© and related cognitive interviews may be limited to patients with undetermined sleep disturbance. Another potential limitation could be that because the objective of the study was to establish content validity, the CSD-AD© and SD NRS were completed at the time of the cognitive interviews and not immediately after sleep disturbance (as intended for both the CSD-AD© and the SD NRS) or in the evening (as intended for the CSD-AD©). This may have resulted in memory bias or an increased difficulty in answering some items. However, it should not have affected the overall conclusions about content validity of the CSD-AD© or SD NRS.

To evaluate the effect of AD treatments in clinical settings and capture the multidimensionality of the AD-related sleep disturbance concepts, we recommend using the SD NRS in conjunction with the CSD-AD©. Nightly self-reported sleep in a diary is regarded as the “gold standard” for the subjective assessment of sleep and tracking of sleep disorders [11]. A potential disadvantage of the CSD-AD© and SD NRS is that they require daily reporting, which could result in a heavy administrative burden or the possibility of missing an assessment. To limit this and possible memory bias due to delayed reporting, for clinical trials, the CSD-AD© and SD NRS will be collected using an electronic device with a reminder or alarm function.

## Conclusions

This qualitative study showed that sleep disturbance is a relevant, multi-dimensional concept that is important, is significant to patients with AD, and can be assessed using self-reported measures. The study further showed that the CSD-AD© and SD NRS yield relevant and meaningful data about AD-related sleep disturbance, and it supports the use of these instruments in clinical trials evaluating the effects of treatments in this population, although their psychometric measurement properties and responder thresholds remain to be established.

## Supplementary Information

**Additional file 1.**

## Data Availability

The data that support the findings of this study are available from Galderma, but restrictions apply to the availability of these data and, so, are not publicly available. Data are however available from the authors upon reasonable request and with permission of Galderma.

## Abbreviations

AD: Atopic dermatitis
CSD-AD: Consensus Sleep Diary, atopic dermatitis version
EASI: Eczema Area and Severity Index
PRO: Patient-reported outcome
SCORAD: SCORing Atopic Dermatitis
SD NRS: Sleep disturbance numerical rating scale
VAS: Visual analogue scale
