# Correction to: Content validity of a sleep numerical rating scale and a sleep diary in adults and adolescents with moderate-to-severe atopic dermatitis

**DOI:** 10.1186/s41687-020-00279-6

**Published:** 2020-12-29

**Authors:** Carla Dias-Barbosa, Rodolfo Matos, Margaret Vernon, Colleen E. Carney, Andrew Krystal, Jorge Puelles

**Affiliations:** 1 Evidera, The Ark, 201 Talgarth Road Hammersmith, London, W6 8BJ UK; 2 Evidera, 7101 Wisconsin Avenue, Suite 1400, Bethesda, MA 20814 USA; 3 Ryerson University, 350 Victoria Street, Toronto, ON M5B 2K3 Canada; 4 University of California, San Francisco, Weill Institute for Neurosciences, 401 Parnassus Avenue, San Francisco, CA 94143-0984 USA; 5 Galderma, World Trade Center, Avenue Gratta-Paille 2, 1018 Lausanne, Switzerland

**Correction to: J Patient Rep Outcomes 4, 100 (2020)**

**https://doi.org/10.1186/s41687-020-00265-y**

Following publication of the original article [1], the authors identified an error in the listed corresponding author. She should be identified as Carla Dias-Barbosa and not as Rodolfo Matos.
