# Supplementary material for: Content validity of a sleep numerical rating scale and a sleep diary in adults and adolescents with moderate-to-severe atopic dermatitis
Source: J Patient Rep Outcomes. 2020 Nov 23;4:100. doi: 10.1186/s41687-020-00265-y (PMC7683746; doi:10.1186/s41687-020-00265-y)
Supplement: Supplementary file 1 — Additional file 1. [file 41687_2020_265_MOESM1_ESM.docx]

# Appendix B. Supplemental Files

## Revised Version of the CSD-AD^©^


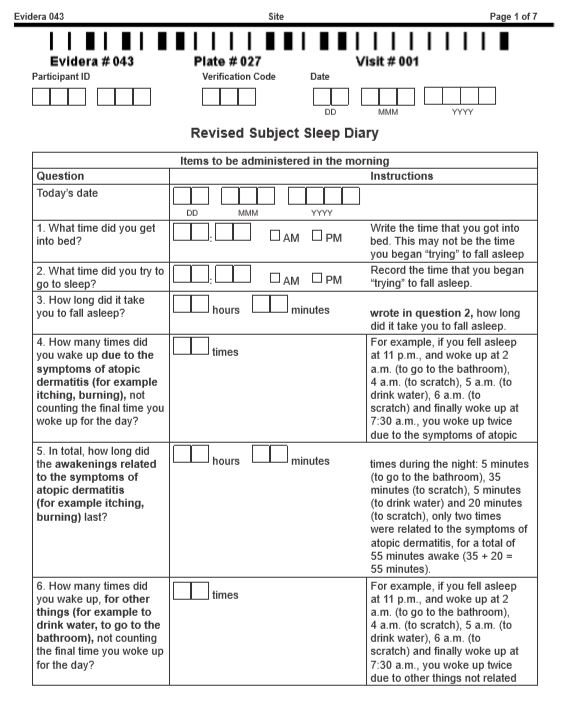


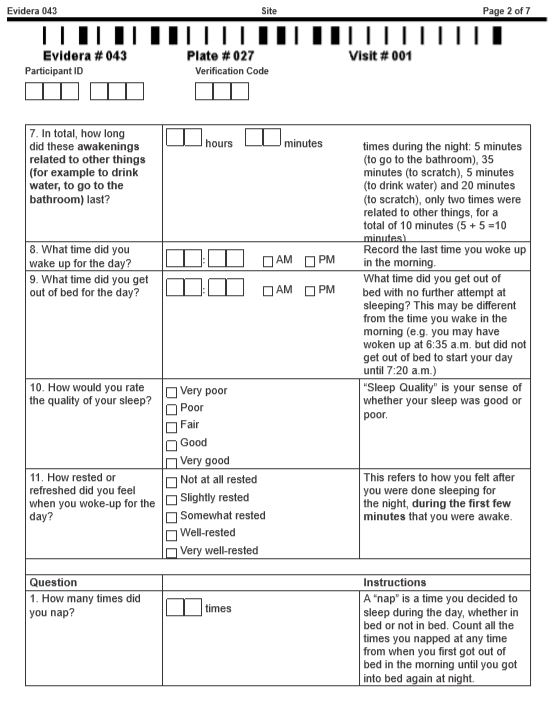


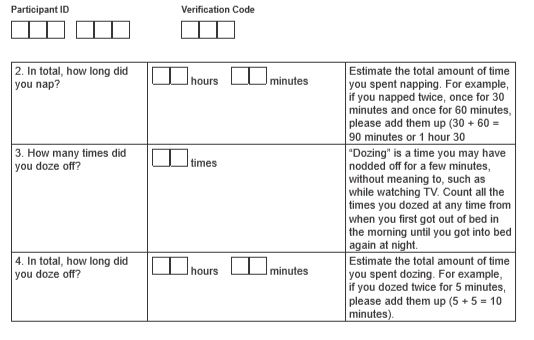


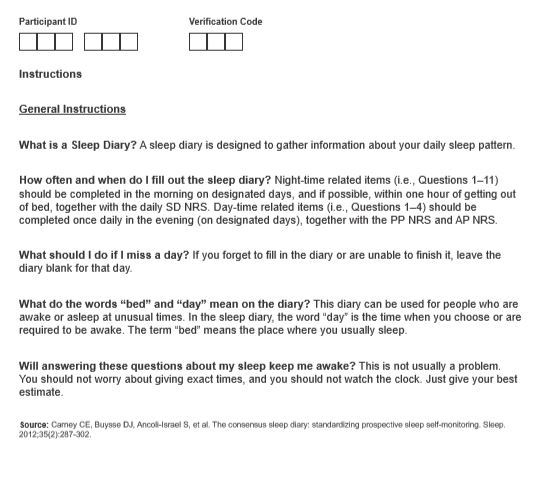


Abbreviations: AP NRS = average pruritus numerical rating scale; PP NRS = peak pruritus numerical rating scale SD NRS = sleep disturbance numerical rating scale
